# Albinism in the domestic cat (*Felis catus*) is associated with a *tyrosinase* (*TYR*) mutation

**DOI:** 10.1111/j.1365-2052.2005.01409.x

**Published:** 2006-04

**Authors:** DL Imes, LA Geary, RA Grahn, LA Lyons

**Affiliations:** Department of Population Health and Reproduction, School of Veterinary Medicine, University of CaliforniaDavis, Davis CA, USA

**Keywords:** albinism, cat, *tyrosinase*

## Abstract

Albino phenotypes are documented in a variety of species including the domestic cat. As albino phenotypes in other species are associated with *tyrosinase* (*TYR)* mutations, *TYR* was proposed as a candidate gene for albinism in cats. An Oriental and Colourpoint Shorthair cat pedigree segregating for albinism was analysed for association with *TYR* by linkage and sequence analyses. Microsatellite *FCA931*, which is closely linked to *TYR* and *TYR* sequence variants were tested for segregation with the albinism phenotype. Sequence analysis of genomic DNA from wild-type and albino cats identified a cytosine deletion in *TYR* at position 975 in exon 2, which causes a frame shift resulting in a premature stop codon nine residues downstream from the mutation. The deletion mutation in *TYR* and an allele of *FCA931* segregated concordantly with the albino phenotype. Taken together, our results suggest that the *TYR* gene corresponds to the *colour* locus in cats and its alleles, from dominant to recessive, are as follows: *C* (*full colour*) > *c*^*b*^ (*burmese*) ≥ *c*^*s*^ (*siamese*) > *c* (*albino*).

Albinism is a congenital disorder that is characterized by lack of pigment in hair, skin and eyes. Recently, the causative mutations for the *siamese* and *burmese* temperature-sensitive alleles have been identified in *tyrosinase* (*TYR*; Lyons *et al.* 2005). Complete albinism in cats is hypothesized to be caused by an additional allele at *TYR*, contributing to the allelic series at the *colour* (*C*) locus: *C* (*full colour*) > *c*^*b*^ (*burmese*) ≥ *c*^*s*^ (*siamese*) > *c* (*complete albino*). To confirm that albinism is a *TYR* allele in cats, an analysis of an Oriental and Colourpoint Shorthair cat pedigree that segregates for albinism (Fig. 1) was tested for linkage with *FCA931*, a marker ∼1.7 cM from *TYR* (Menotti-Raymond *et al.* 1999, 2003). In addition, sequence analyses of *TYR* were conducted to identify a causative mutation for feline albinism.

**Figure 1:**
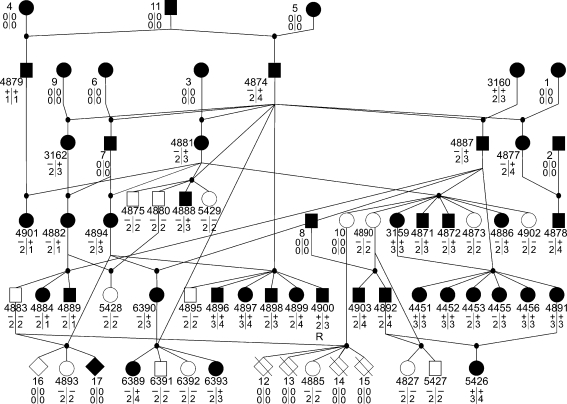
Pedigree segregating for complete and temperature-sensitive albinism (*siamese*, *c*^*s*^) in Colourpoint Shorthair cats. Circles represent females, squares represent males, solid symbols indicate phenotypically siamese-coloured (*c*^*s*^*c*^*s*^ or *c*^*s*^*c*) cats and clear symbols indicate albino cats. Diamonds represent individuals with unknown gender, a slash through a symbol implies the individual was stillborn. Small filled circles represent ‘breeding nodes’ for parental cats. Numbers under the symbols represent laboratory sample numbers. Samples from cats 1–17 were not available for testing. Genotypes for the *C* deletion at position 975 in exon 2 are presented below the laboratory numbers. A ‘+’ indicates the normal wild-type sequence and ‘−’ indicates the *C* deletion. Genotypes for the linked microsatellite marker *FCA931* are represented below the mutation genotypes. The base-pair sizes of the microsatellite markers were converted to single numbers to distinguish the alleles. Missing data are represented by ‘0’. An *‘R’* indicates the detectable recombination event in individual 4900.

DNA was isolated from buccal cells and blood samples from cats in the multi-generational pedigree (Fig. 1) according to published procedures (Sambrook & Russell 2001; Oberbauer *et al.* 2003). Phenotypes were verified by visual inspection, breeder reports, segregation in families and photographs (Fig. 2). Relationship of the cats was verified by parentage testing with 19 microsatellites (data not shown). Pigmented cats that produced albino offspring were assumed to be obligate carriers of the complete albinism allele. Microsatellite *FCA931*, which is linked to *TYR* (Menotti-Raymond *et al.* 1999, 2003), was genotyped as previously described (Grahn *et al.* 2004), and the alleles were tested for concordant segregation with the colour phenotypes.

**Figure 2:**
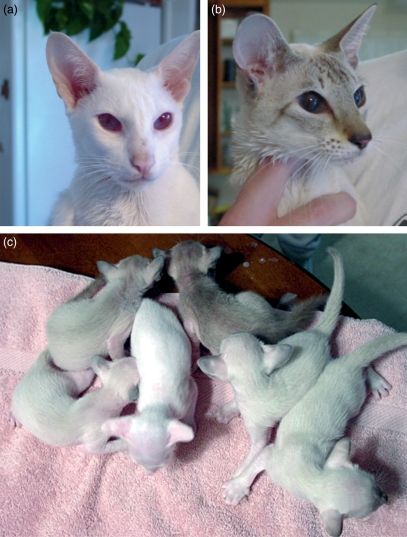
Phenotypes in a domestic cat pedigree (Fig. 1) that segregated for albinism: (a) albino, (b) a Colourpoint (chocolate lynx-point non-albino) sibling ; and c) a litter of kittens that includes an albino (third from the left).

Tyrosinase exons were sequenced as previously described (Lyons *et al.* 2005) from three albino cats and three obligatory carriers, as well as from three wild-type cats that were not associated with the albino pedigree. The *TYR* sequences of the albino cats and the wild-type cats were identical except for a cytosine deletion at position 975 in exon 2, which causes a frame shift and a premature stop codon in the protein translation nine codons downstream of the deletion (Fig. 3). The sequence of the deletion allele was submitted to GenBank (AY743347).

**Figure 3:**
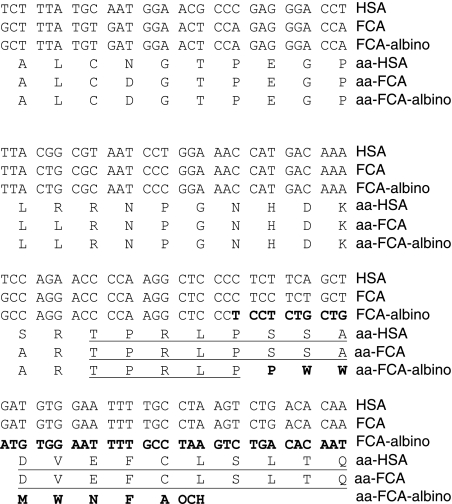
Exon 2 nucleotide and protein sequence alignments of feline and human *tyrosinase* (*TYR*). The *TYR* nucleotide sequences for *Felis catus* (FCA) and *Homo sapiens* (HSA) were AY743347 and M27160 respectively. The amino acids (aa's) for each codon are listed below the nucleotide sequences. The *albino* mutation is a cytosine deletion at nucleotide 975 that causes a frameshift, leading to a stop (OCH) codon nine residues downstream of the deletion GenBank accession no. AY743347. The portion of the cat *albino* allele that is altered relative to the wild-type sequence is presented in bold. Amino acids that are conserved among dogs, human, mice and cattle are underlined. The rabbit has a single amino acid change in this region, replacing an alanine with a serine.

Fifty of 67 cats in the pedigree (Fig. 1) were then screened for the identified deletion mutation by direct sequencing. The genotypes for marker *FCA931* and for the mutation are presented in Fig. 1. The cytosine deletion was homozygous in all 15 albino cats and heterozygous in all seven obligate carriers. Analysis of the pedigree suggested that albinism had an autosomal recessive mode of inheritance. One recombination event was detected between *FCA931* and the colour phenotype. The founder cats were Colourpoint Shorthairs, so these cats have at least one *siamese* allele (*c*^*s*^), which was confirmed by sequence and restriction fragment length polymorphism (RFLP) analyses (Lyons *et al.* 2005).

In our previous study (Lyons *et al.* 2005), mutations in *TYR* associated with the temperature-sensitive phenotypes of siamese and burmese cats were identified. Robinson (1991) suggested that an allele in *TYR* causes albinism because other species have the same phenotype associated with *TYR* mutations. Our analysis of an extended pedigree supports that the albinism phenotype is allelic to *full colour* (*C*), *burmese* (*c*^*b*^) and *siamese* (*c*^*s*^), suggesting the allelic series *C* > *c*^*b*^ ≥ *c*^*s*^ > *c* based on mutations in *TYR*. However, sufficient breeding studies have not been performed to confirm the allelic series in cats, specifically, the interaction of *full colour* and *burmese* with the *albino* allele.

The putative *albino* mutation identified in this study would produce a truncated protein because a stop codon occurs in exon 2, nine amino acids downstream of the deletion. The amino acids located near the cytosine deletion are conserved among dogs, human, mice, cattle and rabbits (Fig. 3), further supporting that this mutation is a significant change in the protein. Expression studies and complementary DNA sequencing are needed to support this finding.

Albino cats have been reported in the literature (Todd 1951; Turner *et al.* 1981) but have not been well characterized. Blue-eyed vs. pink-eyed albino cats have not been clearly distinguished in the published reports (Bamber & Herdman 1931; Todd 1951; Leventhal 1982; Leventhal *et al.* 1985). Thus, it is unclear whether there is more than one non temperature-sensitive albinism allele in cats, as has been reported in mice (reviewed in Beermann *et al.* 2004), in humans (summarized at http://albinismdb.med.umn.edu/) and in cattle (Schmutz *et al.* 2004). The albino cats evaluated in this study have blue eyes. As with most blue-eyed cats, reduced pigment in the tapetum produces a ‘reddish’ (as opposed to a ‘greenish’) tapetal reflection or ‘eye-shine’. The *c* allele has been reserved for red-eyed (complete) albinism, but the difference in the tapetal reflex suggests that the single report of a red-eyed albino cat may be in error.

In conclusion, we propose that a cytosine deletion in *TYR* at position 975 in exon 2 is associated with albinism in cats. This mutation could be used in a DNA test to detect carriers and assist with breeding programmes. This finding also supports the use of the cat as a model for human *TYR*-associated albinisms.
